# Implementation of the Quebec mental health reform (2005–2015)

**DOI:** 10.1186/s12913-016-1832-5

**Published:** 2016-10-18

**Authors:** Marie-Josée Fleury, Guy Grenier, Catherine Vallée, Denise Aubé, Lambert Farand, Jean-Marie Bamvita, Geneviève Cyr

**Affiliations:** 1 Department of Psychiatry, McGill University, 845 Sherbrooke Street, Montreal, H3A 0G4 Quebec Canada; 2 Douglas Mental Health University Institute Research Centre, 6875 LaSalle Blvd., Montreal, Quebec H4H 1R3 Canada; 3 Rehabilitation Department, Laval University, Quebec, Quebec GIV 0A6 Canada; 4 Department of Social and Preventive Medicine, Laval University, National Public Health Institute of Québec, Quebec, Quebec GIV 0A6 Canada; 5 Department of Health Administration, Policy and Evaluation, School of Public Health, University of Montreal, Montreal, Quebec H3T 3J7 Canada

**Keywords:** Mental health reforms, Implementation, Primary care, Networks, Integration, Strategies, Determinants, Shared-care, Collaborative care

## Abstract

**Background:**

This study evaluates implementation of the Quebec Mental Health (MH) Reform (2005–2015) which aimed to improve accessibility, quality and continuity of care by developing primary care and optimizing integrated service networks. Implementation of MH primary care teams, clinical strategies for consolidating primary care, integration strategies to improve collaboration between primary care and specialized services, and facilitators and barriers related to these measures were examined.

**Methods:**

Eleven Quebec MH service networks provided the study setting. Networks were identified in consultation with 20 key MH decision makers and selected based on variation in services offered, integration strategies, best practices, and geographic criteria. Data collection included: primary documents, structured questionnaires completed by 25 managers from MH primary care teams and 16 respondent-psychiatrists working in shared-care, and semi-structured interviews with 102 network stakeholders involved in the reform. The study employed a mixed method approach, triangulating the three data sources across networks.

**Results:**

While implementation was not fully achieved in most networks, the Quebec reform succeeded in improving primary care services with the creation of adult primary care teams, and one-stop services which increased access to care, mainly for clients with common MH disorders. In terms of clinical strategies implemented, the functions provided by respondent-psychiatrists had a greater impact on the MH primary care teams than on general practitioners (GPs) in medical clinics; whereas the implementation of best practices were indirect outcomes of another reform developed simultaneously by the Quebec substance use disorders program. The main integration strategies used for increasing continuity of care and collaboration between primary care and specialized services were those involving fewer formal procedures such as referrals between teams and organizations. The lack of operational mechanisms and protocols governing new services and structures were important barriers to primary care consolidation and service integration, as was the lack of interest and involvement of most GPs in MH.

**Conclusions:**

Successful and sustained healthcare reform requires attention to process and outcomes as well as structural change or service reorganization. Six recommendations for more successful implementation of the Quebec MH Reform are provided, with implications for healthcare reform internationally.

**Electronic supplementary material:**

The online version of this article (doi:10.1186/s12913-016-1832-5) contains supplementary material, which is available to authorized users.

## Background

Mental health disorders (MHD) are a leading cause of worldwide health-related disability [1]. Depression is predicted to become the greatest contributor to global disease burden by 2030 [2]. MHD co-occur with medical disorders such as diabetes, cardio-vascular disease and substance use disorders (SUD), and with social problems such as poverty and victimization [2]. Yet relatively few affected individuals use mental health (MH) services [3, 4]. With fragmented services identified as a critical barrier to care [5], the MH needs of individuals can best be met through a range of continuous, diversified and integrated bio-psycho-social services [5].

Most industrial countries including the US, UK and Australia have reformed their MH systems over the past two decades, generally aiming to improve access, quality and continuity of care [6–8]. Treatment has shifted from hospital to community [9, 10], reinforcing primary MH care, and integrating primary care with specialized MH services, including SUD treatment [11, 12]. Interest in providing evidence-based practices such as assertive community treatment (ACT) or cognitive behavioral therapy is strong [13]. Integration strategies at administrative and clinical levels have also been found to facilitate both reform implementation and organizational integration [14]. Although MH reforms encompass similar objectives, they differ in terms of timing, problems addressed, and the structures or clinical interventions implemented [15]. For example, reform in Finland included SUD as a MH disorder [16]. MH reform in England clearly specified the desired functions, operations and outcomes [7]; whereas reform in Belgium focused more on structures targeted for implementation [10].

In this context, the Quebec (Canada) Ministry of Health and Social Services (henceforth Quebec Ministry) developed a mental health action plan (henceforth MH Reform) in 2005 that mandated a major reorganization of services and strengthening of primary MH care [17]. The MH Reform resulted from broad consultation with 500 Quebec MH stakeholders [17]. Key issues that sparked the reform included long wait times for psychiatric care, insufficient services for common MHD (anxiety and depression) due in part to the reluctance of general practitioners (GPs) to accept MH cases, and an underperforming system insufficiently attuned to client recovery and quality care.

Within the Quebec public healthcare system, prescription drugs are provided free of charge, as are MH services excluding those provided by psychologists in private practice. MH specialized services are offered in psychiatric or general hospitals, according to regional variation in conditions and service availability; and primary care services in public local health service centers or medical clinics. Community organizations (crisis centers, peer and family self-help groups, etc.), residential resources, and inter-sectorial resources including SUD rehabilitation centers complete the Quebec MH system. Physicians (GPs or specialists) are generally paid on a fee-for-service basis, with the exception of a minority of GPs working in local health service centers, who are salaried professionals. The ratio of GPs per inhabitants in Quebec (1,03 per 1000) and in Canada (1,12 per 1000) is above average for industrial countries [18]. Moreover, 21 % of the Quebec population does not have a GP or family physician to assume continuity of care [19]. Although GPs are the first point of contact in the Quebec healthcare system for clients with MHD, this does not necessarily translate into adequate quality of services or continuity of care for this population [20, 21].

Quebec healthcare services are integrated with social services and managed at the provincial, regional, and local levels. The Quebec Ministry is responsible for overall governance and control of healthcare. Within each of 15 provincial regions, a regional agency establishes budgets and coordinates the local Health and Social Service Networks. In the context of a more global reform of the Quebec healthcare system that occurred simultaneously in 2005, 95 local service networks were created, each with a Health and social service center (HSSC) emanating from the merger of acute care hospitals, nursing homes and local community service centers. HSSCs are responsible for service integration and quality care in MH, and other healthcare programs within their respective networks (before April 2015). The SUD program for example had initiated training in HSSCs, using standardized tools for SUD identification, screening and early intervention, as well as motivational interviewing. SUD specialists offered expertise and advice to HSSC clinicians, while emergency-liaison teams worked to reduce the overflow of individuals with SUD or co-occurring MHD-SUD in emergency rooms [22].

In order to increase access to MH services, the MH Reform proposed measures to enhance primary care. Each HSSC was mandated to create one or more MH primary care teams for treating common MHD among adults. Regarding staff requirements for the new HSSC-MH adult primary care teams, the MH Reform projected 20 full time psychosocial clinicians and two GPs per 100 000 inhabitants. Moreover, for networks with 50 000 or more inhabitants, a one-stop service was set up as the point of entry for accessing MH services, whether supplementary MH services for individuals under the care of GPs, or patient referrals from hospitals to specialized outpatient services [17]. Clients referred by GPs, community organizations, inter-sectorial resources, as well as stabilized patients in specialized MH services ready for transfer to primary care, obtained clinical MH assessments at the one-stop services. A maximum 7 day wait time for MH assessment, and 30-day wait time from MH assessment to treatment were projected [17]. Most HSSC-MH adult primary care teams became operational by 2008, and MH one-stop services by 2009 [23].

In order to improve quality of care, the MH Reform promoted recovery best-practices (e.g. care pathways, cognitive behavioral therapy) [17]. For clients with severe MHD in particular, community support programs such as intensive case management were established under the direction of HSSC-MH primary care teams or community organizations. Research has demonstrated the effectiveness of best-practices for improving outcomes among clients with MHD [24–31]. Shared-care was also promoted by hiring respondent-psychiatrists (3 h per months per 50 000 inhabitants) to provide consultation and support to HSSC-MH primary care teams and GPs [17]. According to a meta-analysis, shared-care improves the capacity of GPs to prescribe pharmacological therapy and to provide adequate treatment [32]. Individuals with common MHD, particularly depression, experienced greater satisfaction and adherence to treatment [32, 33]; whereas the effectiveness of shared-care has yet to be demonstrated for severe and co-occurring MHD-SUD. The respondent-psychiatrist position was only ratified in 2009 after protracted negotiation between the Quebec Psychiatric Association and the Quebec Ministry; the first respondent-psychiatrists were appointed the following year. Furthermore, the MH Reform promoted better collaboration between primary and specialized MH services. Integration strategies, such as service agreements and use of liaison officers, providers who relay information between services or organizations serving the same clientele, were advanced and proved effective for MH providers [34].

The reform implementation process marks a critical phase generally, and entails a high risk of failure [35]. Barriers to implementation reported in the literature include lack of financial or human resources [36, 37], staff turnover [38], absence of leadership [39], obstacles to information exchange among professionals [40], poor inter-organizational collaboration [41], professional resistance to new working cultures [11], and lack of role clarity [42]. With these issues in mind, the present study evaluates: 1) implementation of the HSSC-MH adult primary care teams in terms of their impact on access to care for different client populations; 2) implementation of key clinical strategies, including shared-care, clinical approaches (e.g. cognitive behavioral therapy) and clinical evaluation tools (e.g. screening tools for MHD, Table 1), for their potential to consolidate primary care and improve service quality; 3) implementation of integration strategies aimed at improving collaboration, continuity and integration between primary care and specialized services; and 4) facilitators and barriers to primary care consolidation and network integration. This study is original in evaluating a comprehensive, system-wide reform that undertook a major shift toward primary care and strengthening of integrated service networks, while introducing shared care and other recognized best practices. These targets are central to most international MH reforms in advanced healthcare systems.

**Table 1:** Analytical framework and synthesis of the MH Reform implementation targets

Objective 1: Consolidation of the Health and Social Services Centers (HSSC) and Mental health (MH) primary care teams (HSSC-MH primary care teams) for the 11 local service networks under study				
Quebec MH Reform targets	Not achieved	Partially achieved	Achieved	N/A
HSSC-MH adult primary care teams				
20 multidisciplinary MH clinicians/100 000 inhabitants	6		5	
2 general practitioners (GPs)/100 000 inhabitants * For a given network, achieved in some, but not all teams	9	1*	1	
Access to treatment: 30 days	7		3	1
MH one-stop services				
A MH one-stop service in all networks with a population of 50 000 + inhabitants * In one network, staffing incomplete		1*	9	1
Access to evaluation: 7 days	5		5	1
Objective 2: Strategies used to consolidate primary care and improve quality of care				
a) Consolidation of the respondent-psychiatrist function in the 11 networks under study				
Quebec MH Reform targets	Not achieved	Partially achieved	Achieved	N/A
1 respondent-psychiatrist/50 000 (3 h/service per months: to HSSC-MH teams and GPs)	3		8	
b) Intensive case management				
Intensive case management in HSSC * Required number of teams not achieved in 4 networks		4*	7	
Intensive case management offered by MH community organizations (but under the responsibility of the HSSC)	6		5	
c) Clinical approaches & clinical evaluation tools based on the literature [67]				
Clinical approaches (Best practices) 7 approaches	Stepped-care: Care delivery model in which interventions are performed hierarchically based on the intensity of client problems. Mainly effective for depression [25].			From high to moderate use (See Table 4)
	Cognitive behavioral therapy: Psychotherapy aiming to change thinking and behavior. Effective for most mental health disorders, including SUD [26].			
	Motivational interviewing: Brief intervention aiming to engage motivation to change behavior. Mainly effective for substance use disorders [27].			
	Strengths model: Intervention focusing on the strength and interests of the user rather than pathology, and oriented toward achieving goals set by the user him/herself. Mainly effective for severe mental health disorders [28, 68].			
	Care pathways: Systematic interventions planned for integrating care between different organizational units or between providers for a well-defined group of clients and treatment periods. Originally established for acute medical care, for which it has been proven effective. This care process aims at enhancing continuity of care and system efficiency, and is also applied currently in MH [29].			
	Self-management: Systematic provision of education and supportive interventions in order to increase skills and confidence of clients in managing their health problems. Mainly effective for depression [30].			
	Recovery approach: Personal journey that involves developing a secure sense of self, supportive relationships, empowerment, social inclusion, coping skills, and new meaning in life. In most longitudinal studies, recovery rates were 80 % for bipolar disorders, 65 to 80 % for major depression, 70 % for substance disorders and 60 % for schizophrenia [31, 69].			
Clinical evaluation tools: establish clinical standardization and rationalization to promote best practices [14].	• Screening tools for MHD			From high to low use (See Table 4)
	• Screening tools for SUDs			
	• Assessment tools for MHD			
	• Assessment tools for SUDs			
	• Assessment tools for client satisfaction			
	• Clinical protocols and best practice guidelines			
Objective 3- Strategies used to increase network integration (coordination between primary care and MH specialized services in each network)				
Integration strategies 10 key strategies	Liaison officer: Professional designated by an organization to relay information between departments of the same organization, or between organizations serving the same clientele [14].			From many to few implemented (See Table 4)
	Shared training: A strategy to enhance collaborative environments by simultaneously training clinicians with different areas of expertise, and/or from different services or organizations in a network [70].			
	Shared staff: Professionals offering services across more than one organization to insure coverage of the required range of services and to intensify inter-organizational collaborations [14].			
	Service agreement: Administrative strategy used in formalizing mechanisms that facilitate access and continuity of services between at least two organizations, or between programs in the same organization [14].			
	Referral mechanisms: • Shared clinical records • Network resources directory • Referral procedures within organizations • Referral procedures between organizations			
	SUD specialist respondent: Specialist in SUD who holds case discussions with MH and other teams concerning SUD, aiming to reinforce SUD expertise and interventions for both SUD and co-occurring MHD-SUD.			
	Individualized service plans: Mutual agreements among service providers, the client or his/her representative (or family) defining which care or service objectives to pursue. Plans usually target clients with multiple and often severe needs, who require case coordination involving several providers [71].			Not included in Table 4

## Methods

### Study design and data collection

The study employed a mixed method approach, triangulating data sources across 11 of the 95 Quebec MH service networks. Networks were identified for the study in consultation with a research advisory committee composed of 20 Quebec MH decision makers (e.g. the MH director in the Quebec Ministry, MH regional coordinators, a representative of the Quebec Psychiatric Association) who completed a survey. The 11 networks were selected for maximum variation and representativeness in terms of geographic area (urban, semi-urban, and rural), the organization of primary and specialized care, presence or absence of a psychiatric hospital; and perceived levels of implementation of the MH plan (from high to low). The evaluation by the research advisory committee also took into account a number of other factors including the range of services offered, integration strategies developed (e.g. services agreements, liaison officers), uptake of best-practices (e.g. cognitive behavioral therapy, motivational interviewing), and barriers and facilitators associated with the implementation process.

Data were collected from three sources: 1) structured questionnaires completed by managers from HSSC-MH primary care teams, including one-stop services and intensive case management services, and by respondent-psychiatrists; 2) semi-structured interviews with key network stakeholders involved in the reform; and 3) primary documents written by managers on issues related to MH teams, organizations and networks. For each network, all managers of HSSC-MH primary care teams and respondent-psychiatrists were invited to complete questionnaires. The great majority of managers did so after consulting with their teams, and with available data banks. Key stakeholders from the 11 networks were invited to participate in the individual qualitative interviews or focus groups (Table 2). The number of interview participants was based on network size. The research advisory committee helped with data collection. Questions for both the structured questionnaires and qualitative interview guides were developed and customized for this study as is usual for all descriptive and exploratory research on organizations. The questionnaires and interview guides were pretested with three participants respectively, and validated by the research advisory committee.

**Table 2:** Socio-demographic description of professionals

	Managers/Coordinators of MH^b^ services (*N* = 25)	Respondent- psychiatrists (*N* = 16)	Interviews (*n* = 102)	Total (*n* = 143)
Average age [Mean (SD)]	42.9 (8.7)	49.1 (10.5)	50.7 (8.8)	49.1 (9.4)
Gender [n (%)]				
Female	19 (76.0)^a^	6 (37.5)	69 (67.6)^a^	95 (66.0)^a^
Male	6 (24.0)	10 (62.5)^a^	33 (32.4)	49 (34.0)
Current position [n (%)]				
Psychiatrist	–	16 (100.0)	7 (6.9)	23 (16.1)
General practitioner	–	–	10 (9.8)	10 (9.8)
Psychosocial clinician	–	–	4 (3.9)	4 (3.9)
Regional Manager	11 (44.0)	–	4 (3.9)	15 (10.5)
Director	–	–	35 (34.3)	35 (34.3)
Program Administrator/Coordinator	14 (56.0)^a^	–	42 (41.2)^a^	56 (39.2)^a^
Years of experience [Mean (SD)]				
In the current position (in years)	5 (7.1)	2.9 (5.0)	7.9 (6.7)	5.3 (6.2)
In psychiatry	–	17.8 (10.7)	–	17.8 (10.7)
In the health and social science	–	–	23,1 (8,6)	23,1 (8,6)
In mental health	–	–	19.4 (9.3)	19.4 (9.3)
In adult mental health	–	–	19.5 (9.3)	19.5 (9.3)
Organization [n (%)]				
Regional agency	–	–	11 (10.8)	11 (10.8)
Psychiatric hospital	–	–	14 (13.7)	14 (13.7)
General hospital (GH)	–	–	9 (8.8)	9 (8.8)
Health and social service center	25 (100.0)^a^	–	44 (43.1)^a^	69 (48.3)^a^
Medical clinic	–	–	7 (6.9)	7 (6.9)
Community organization	–	–	17 (16.7)	17 (16.7)
Territorial profiles [n %]				
With a psychiatric hospital	13 (52.0)^a^	4 (25.0)	37 (36.3)^a^	54 (37.8)^a^
Without specialized MH Services	2 (4.0)	1 (6.3)	16 (15.7)	19 (13.3)
< 200 000 inhabitants, with a psychiatric department in a GH	6 (30.0)	2 (12.5)	21 (20.6)	29 (20.3)
> 200 000 inhabitants, with a psychiatric department in a GH	4 (20.0)	9 (56.3)^a^	28 (27.4)	41 (28.7)

^a^Most important group

^b^Mental health

Primary documents obtained between November 2012 and March 2013 provided data on population and MH service characteristics, and on integration strategies, dynamics, and related challenges for each network. Questionnaires completed between October 2013 and June 2014 were self-administered, and included categorical and continuous items with five- or six-point Likert scale responses. The questionnaire for HSSC-MH primary care teams covered several dimensions: 1) client characteristics (e.g. age, gender, diagnosis), 2) team profiles (e.g. number and type of professionals), 3) clinical activities (e.g. time allocated to evaluation, treatment or intervention), 4) network integration strategies (e.g. shared training, service agreements), and 5) frequency and satisfaction of interactions involving network teams or organizations (e.g. emergency departments, hospital units). The respondent-psychiatrist questionnaire covered three topics: 1) client characteristics (e.g. age, diagnosis), 2) respondent-psychiatrist activities and time allocations (e.g. visits to medical clinics, case discussions), and 3) respondent-psychiatrist impact on MH services (e.g. GP skills related to patient care, diagnosis). Questionnaires for the HSSC-MH primary care teams, and respondent-psychiatrists, took 120 and 30 min respectively to complete.

Interview guides for the qualitative research phase were developed and adapted to each stakeholder group: regional managers, HSSC-MH primary care teams and senior hospital executives, respondent-psychiatrists, GPs, directors of community organizations. Interviews were conducted between March and June 2014, and addressed issues related to: 1) client characteristics (e.g. “How would you describe the main needs and challenges of your clients?”; 2) implementation of the MH Reform (e.g. “How was the respondent-psychiatrist function implemented in your team, clinic or network?”); 3) MH network integration (e.g. “How would you describe consultation-liaison activities in your territory?”); 4) facilitators and barriers to implementation and to network integration (e.g. “What were the main challenges encountered in implementing the respondent-psychiatrist function and HSSC-MH primary care teams in your territory?”), and 5) recommendations for improving MH services. Individual interviews lasting 30–60 min were conducted, in person or by telephone, by authors G.C. and G.G., while 60–90 min focus groups were conducted in person by G.C. accompanied by one of the team researchers (C.V., D.A., M.-J.F.). Interviews were audio-recorded. Socio-demographic data were collected for all participants; anonymity and confidentiality were upheld. A summary of the main topics covered by the interviews and the questionnaires is provided in the Additional file 1.

### Analyses

Quantitative descriptive analysis using SPSS-17.0 software was used to manage data related to the first three study objectives. Frequency distributions for categorical variables and mean values for continuous variables were computed. Table 1 presents the analytical framework for objectives 1–3, identifying major targets and integration strategies with respect to respondent-psychiatrists, clinical approaches, and clinical evaluation tools for consolidating primary care and integrating service networks.

The qualitative analysis of factors influencing the consolidation of primary care and network integration (objective 4) followed a six-step approach: 1) interview transcriptions; 2) preliminary readings; 3) selection and definition of classification units; 4) development of analytical framework (coding tree); 5) separation of content into units of meaning; and 6) data management with N-Vivo software, version 10 [43]. Coding was based on the aforementioned interview topics, allowing for inclusion of emerging issues. Codes were structured around participant teams, organizations, and networks. Inter-rater reliability was verified for 20 % of the codes. Additional file 2 provides representative quotations from the qualitative interviews.

Three summary reports were produced for the quantitative and qualitative results, as well as the investigation of primary documents respectively. A synthesis document was then compiled integrating results of the summary reports, and serving as the basis for the present article. Data analysis was conducted by G.C. J.-M.B. and G.G. under supervision by the researchers (M.-J.F., C.V., D.A., L.F.).

## Results

### Description of the sample

In all, 28 HSSC-MH primary care team managers and 20 respondent-psychiatrists were recruited for the quantitative phase of the study; 25 managers and 16 respondent-psychiatrists participated for a response rate of 86 % (Table 2). For the qualitative research, 110 key stakeholders were recruited, and 103 participated, for a response rate of 94 %. In all, 78 interviews were conducted; 63 individual interviews and 15 focus groups with a maximum of four participants each. The mean age of study participants was 49 years old. Most were female, and worked as program administrators or coordinators in HSSCs; the average tenure in their current position was 5 years.

### Consolidation of HSSC-MH primary care teams to improve access to services

At least one HSSC-MH adult primary care team was implemented in each network (Table 1), although some urban networks had more. The projected ratio of professionals in the teams per 100 000 inhabitants was realized in six networks (54 %), but the projected ratio of GPs in only one (10 %). A one-stop service was established in 9 of 10 networks in areas with 50,000 or more inhabitants. The 30-day benchmark for access to services after assessment at the MH one-stop services was achieved in only 3 networks (27 %).

The average delay for access to one-stop services was 25 days, versus 89 days for the HSSC-MH adult primary care teams (Table 3). Most one-stop services offered interventions for clients on waiting lists, mainly brief group sessions. Staff in the HSSC-MH primary care teams included social workers, nurses, psycho-educators and psychologists, supported by psychiatrists and SUD specialists. The proportions of clients affected by serious MHD (e.g. schizophrenia, bipolar disorders) and common MHD were nearly equivalent (37 % vs 35 %) in the HSSC-MH primary care teams; 36 % had a co-occurring MHD-SUD; 32 % a personality disorder; 27 % suicidal ideation and 22 % co-occurring MH-chronic physical disorder (e.g. diabetes).

**Table 3:** Composition and activities of HSSC^a^-MH^b^ primary care teams (*N* = 25)

HSSC-MH adult primary care teams (*N* = 14)	Mean	SD
Psychologists	6.8	6.0
Social workers	5.6	4.9
Psycho-educators	4.2	8.4
Nurses	2.9	3.7
Psychiatrists	1.6	4.9
Substance use disorder (SUD) specialists	1.5	4.5
Occupational therapists	1.0	2.1
General practitioners	0.2	0.5
Full time clinicians	19.0	29.1
MH one-stop services (*N* = 5)	Mean	SD
Psychologists	2.8	3.6
Social workers	1.7	2.9
Nurses	1.7	2.0
General practitioners	0.3	0.6
Psychiatrists	0.2	0.4
Psycho-educators	0.0	0.0
Occupational therapists	0.0	0.0
SUD specialists	0.0	0.0
Full time clinicians	3.3	0.4
Intensive case management teams (*n* = 6)	Mean	SD
Psycho-educators	3.8	4.2
Social workers	3.1	2.2
Nurses	2.2	1.9
SUD specialists	0.3	0.5
General practitioners	0.3	0.6
Psychologists	0.2	0.4
Psychiatrists	0.1	0.1
Occupational therapists	0.0	0.0
Full time clinicians	11.5	10.0
Time allocated to treatment or intervention	%	SD
HSSC-MH adult primary care teams (%)	71.1	5.5
MH one-stop service teams (%)	18.0	16.4
Intensive case management teams (%)	58.3	19.1
Time devoted to evaluation	%	SD
HSSC-MH adult primary care teams (%)	15.9	15.4
MH one-stop service teams (%)	46.0	25.8
Intensive case management teams (%)	18.0	8.4
Time allocated to coordination with other teams	%	SD
HSSC-MH adult primary care teams (%)	11.4	6.8
MH one-stop service teams (%)	36.8	26.4
Intensive case management teams (%)	21.7 %	10.3
Delay for access to services	Mean	SD
MH one-stop services (days)	25.0	73.3
HSSC-MH adult primary care teams (days)	89.4	75.8
Frequency of visits by HSCC-MH Adult primary care teams per month	%	SD
2–4 times (%)	51.2	14.5
5 times and more (%)	14.8	7.6
Once (%)	11.1	10.5
< Once (%)	13.9	13.2
Duration of follow-up visits by HSCC-MH adult primary care teams	%	SD
> 1 year (%)	41.6	28.4
< a year (%)	22.5	15.8
< 6 months (%)	13.9	11.9
< 3 months (%)	24.0	14.8
Frequency of follow-up visits by clients of intensive case management teams per month	%	SD
Two times (%)	44.4	25.4
Four times (%)	27.8	18.5
5 times and more (%)	27.8	22.4
Proportion of clientele referred by HSSC-MH adult primary care teams to	%	SD
Specialized MH services (%)	19.1	11.1
MH community organizations	32.4	26.9
Other community organizations	16.0	14.9
Rehabilitation centers	13.9	13.2
Inter-sectorial resources (e.g. education, municipalities)	20.8	8.2
Proportion of clientele referred by MH one-stop services to:	%	SD
Specialized MH services (%)	19.3	14.1
MH community organizations	24.0	19.3
Other community organizations	10.5	10.2
Rehabilitation centers	9.1	5.6
Inter-sectorial resources (e.g. education, municipalities)	5.3	5.5
Proportion of clientele referred by intensive case management teams to:	%	SD
Specialized MH services (%)	30.8	41.8
MH community organizations	46.0	33.6
Other community organizations	36.6	10.1
Rehabilitation centers	7.8	7.7
Inter-sectorial resources (e.g. Education, municipalities)	11.8	6.5

^a^Health and Social Service centers

^b^Mental health

### Implementation of clinical strategies to consolidate primary care and improve quality of services: respondent-psychiatrists, intensive case management teams, clinical evaluation tools

Respondent-psychiatrists were deployed in 8 of the 11 networks (73 %) **(**Table 1 **)**, dedicating more hours per month on average to HSSC-MH primary care teams than to GPs, both in terms of telephone consultations (6 versus 4 h) and face-to-face consultations (5 versus 3 h). Their time allocation on the HSSC-MH primary care teams by specific activities was as follows: case discussion (49 %), treatment recommendation (24 %), diagnostic evaluation (12 %), pharmacological recommendation (9 %), and other clinical activities (6 %). Regarding consultations with GPs, respondent-psychiatrists distributed their time among the following activities: pharmacological recommendation (30 %), case discussions (29 %), diagnostic evaluation (18 %), treatment recommendation (17 %), and other clinical activities (6 %).

Inter-professional collaboration with HSSC-MH primary care teams was reported by respondent-psychiatrists as more satisfactory than collaboration with GPs (81 % vs 63 %). Moreover, 75 % of respondent-psychiatrists rated their impact on the quality of care provided by HSSC-MH primary care teams as high or very high. By contrast, 75 % rated their impact as low to medium in terms of their ability to help GPs with diagnosis, or with the quantity and quality support provided by GPs to clients.

Intensive case management teams were fully implemented in 7 networks, but were contracted out to community organizations in 5 of them. Intensive case management teams serviced clients with severe MHD (77 %, s.d. 17 %) as compared with HSSC-MH adult primary care teams (24 %, s.d. 13 %), and one-stop services (16 %, s.d. 10 %). Many clients served by case management teams were heavy MH service users (43 %, s.d 38 %) as compared with clients in HSSC-MH adult primary care teams (11 %, sd. 14 %) or one-stop services (4 %, s.d. 6 %).

Cognitive behavioral therapy and motivational interviewing (described in Table 1) were the clinical approaches most often used by HSSC-MH primary care teams; whereas stepped-care was less often used (Table 3). Co-occurring SUD screening and assessment were the most frequently used clinical evaluation tools while patient satisfaction assessment tools were less often used by HSSS-MH primary care teams (Table 3).

### Key integration strategies and contacts promoting collaboration and continuity of care between HSSC-MH primary care and specialized MH services

Network resource directories, inter- and intra-organizational referral procedures, and shared clinical records were the most frequently implemented integration strategies (Table 4). Service agreements, liaison officers and shared training were moderately implemented; whereas SUD specialists and shared staff between organizations were least implemented. Individualized service plans were organized for fewer than 20 % of clients.

**Table 4:** Frequency of use of clinical approaches, clinical evaluation tools and integration strategies by HSSC-MH Primary care teams (*n* = 25)

	Minimum	Maximum	Mean	Std. deviation
Clinical approaches				
Cognitive behavioral therapy	1	5	3.32	1.14
Motivational interviewing	1	5	3.20	0.87
Strengths model	1	5	2.96	1.10
Care pathway	1	5	2.92	1.19
Recovery	1	5	2.88	1.09
Self-management	1	4	2.56	0.96
Stepped care	1	5	2.04	1.21
Clinical evaluation tools				
Screening tools for SUD^a^	2	6	3.96	1.27
Assessment tools for SUD^a^	1	6	3.68	1.60
Clinical protocols and best-practice guides	1	5	3.24	1.33
Assessment tools for MHD^b^	1	6	3.12	1.54
Screening tools for MHD^b^	1	6	2.84	1.62
Assessment tools for patient satisfaction	1	6	2.32	1.18
Integration strategies				
Network resource directory	3	6	4.44	0.87
Referral procedure within the organization	2	6	4.32	1.18
Referral procedure between organizations	3	6	4.20	1.12
Shared clinical records	1	6	3.88	1.76
Service agreements	2	5	3.08	0.76
Shared training	1	5	3.00	1.04
Liaison officers	1	5	2.96	1.43
SUD^a^ specialists	1	5	2.44	1.39
Shared Staff	1	4	2.12	1.17

Mean score: minimum = 0; maximum = 5; Higher = greater use

^a^Substance use disorders

^b^Mental health disorders

HSSC-MH primary care teams referred a preponderance of clients to community organizations (e.g. crisis centers, self-help groups) followed by specialized MH services (Table 3). Concerning interactions with other services or teams, HSSC-MH primary care teams interacted with respondent-psychiatrists most frequently (Table 5), followed by GPs in medical clinics, one-stop services for general health and social care, and crisis centers. Their interactions with crisis centers were reportedly most satisfactory, followed by interactions with one-stop service for general health and social care, respondent-psychiatrists, and for SUD rehabilitation centers (Table 5).

**Table 5:** Frequency of interactions, and satisfaction of interactions with other services or organizations among HSSC^a^-MH^b^ primary care teams (*n* = 25)

	Minimum	Maximum	Mean	Std. Deviation
Frequency of interactions				
Responding psychiatrist GH & PH^c^	1	5	3.84	1.41
GPs^d^ in medical clinics	2	5	3.20	1.00
HSSC^a^ one-stop services for general health and social care	1	5	3.12	1.30
Crisis Centers	2	5	3.12	1.13
Community organizations not for MH^b^	1	5	2.80	1.00
SUD^e^ rehabilitation centers	1	5	2.76	1.05
Outpatient clinics GH & PH^c^	1	5	2.74	0.91
Hospital units GH & PH^c^	1	5	2.72	0.97
HSSC^a^ general services	1	5	2.68	1.25
Emergency GH & PH^c^	1	4.5	2.46	0.91
Day hospitals GH & PH^c^	1	4	2.12	0.75
ACT^f^ teams GH & PH^c^	1	5	2.02	0.90
Satisfaction with interactions				
Crisis Centers	2	5	4.00	0.82
HSSC^a^ one-stop services for general health and social care	2	5	3.92	0.86
Respondent-psychiatrists GH & PH^c^	2	4.5	3.88	0.64
SUD^e^ rehabilitation centers	1	5	3.64	0.99
HSSC^a^ general services	1	5	3.60	0.87
Community organizations not for MH^b^	2	5	3.56	0.71
GPs^d^ in medical clinics	2	5	3.36	0.86
Hospital units GH & PH^c^	2	4	3.22	0.65
Emergency rooms GH & PH^c^	2	4	3.04	0.50
Outpatient clinics GH & PH^c^	0	5	2.36	2.04
Day hospitals GH & PH^c^	0	4	1.86	1.37
ACT^f^ teams GH & PH^c^	0	4	1.80	1.42

Mean score: minimum = 0; maximum = 5; Higher = greater use

^a^Health and social service centers

^b^Mental health

^c^General hospitals and psychiatric hospitals

^d^General practitioners

^e^Substance use disorders

^f^Assertive community treatment

### Barriers and facilitators to HSSC-MH primary care consolidation and network integration

The main reported barriers to both HSSC-MH primary care consolidation and network integration (Additional file 2) were the restricted focus of the MH Reform on implementation of new services, while underestimating the importance of operational mechanisms including clinical evaluation tools and integration strategies; and the lack of protocols to determine team roles in primary and specialized care, services offerings and client eligibility. Other barriers were insufficient funding to complete the HSSC-MH primary care teams; high staff turnover and resistance to change; and lack of interest in MH among GPs. Barriers involving the integration of respondent-psychiatrists included: poorly defined roles; negative perceptions among GPs regarding the usefulness of respondent-psychiatrists; absence of financial incentives for GPs as opposed to respondent-psychiatrists; and concerns among psychiatrists that their time commitments to the respondent-psychiatrist function would increase wait lists in MH specialized services. Finally, other barriers concerned network integration efforts, including strong “hospital-centrism” in some networks, and apprehensions among community organizations around developing service agreements with public institutions such as HSSCs for fear of losing their autonomy.

The MH National Center of Excellence, an agency created within the Quebec Ministry to support the reform, was perceived as a strong facilitator of both primary care consolidation and service integration. The MH National Center of Excellence assisted with operationalizing MH one-stop services, selecting standardized tools, and developing intensive case management teams while playing a leadership role in service integration for some networks. Yet the liaison officers were most frequently cited for promoting network integration. While lack of funding was considered a barrier to completing the HSSC-MH primary care teams, this factor was also mentioned in certain networks as an important driver of service integration. That is, resource scarcity forced organizations to be creative in finding ways to work collaboratively in the face of pressing demands. In addition, patient-centered and needs-based philosophies were reported as enabling factors for service agreements between MH primary and specialized care, furthering network integration. The physical proximity of organizations or professionals also served as facilitators for improving collaboration and service continuity. Finally, the leadership role played by the HSSCs, combined with a willingness to collaborate among stakeholders, helped break down silos and foster service integration.

## Discussion

The objectives of the Quebec MH Reform were similar to those characterizing reforms undertaken in other industrialized countries. The Quebec reform aimed to improve: 1) access to MH services especially by reinforcing primary care; 2) quality of care through the development of shared care and best-practices and 3) continuity of care through better integration of primary care with specialized MH services. Overall, the results of this study demonstrate that the objectives of the MH Reform were not fully met. Concerning the first objective, i.e. access to care, standards for accessing treatment through the one-stop services, as well as quotas of psychosocial professionals and GPs in HSSC-MH adult primary care teams, were not attained in most networks. The fact that the Quebec MH Reform focused mainly on the implementation of new structures, and less on operational mechanisms, may explain the difficulties encountered in implementing the one-stop services or HSSC-MH adult primary care teams in some networks. This emphasis on structure seems to reflect the division of authority in the Quebec health system: while the Quebec Ministry provides the main orientations, regional agencies are responsible for budgeting and coordination in their networks, and local territories led by the HSSCs manage operational modalities. As a result, local territories with relatively few material or human resources or without strong leadership had greater difficulty implementing new services and practices. Furthermore, regional and local network regulations are generally more consultative than hierarchical or top-down [44]. Yet the literature highlights the need for centralized network structures, clear guidelines and benchmarks as well as stakeholder support in implementing complex interventions such as those advanced by the Quebec MH Reform [10, 45]. The focus on structure rather than implementation processes or outcomes was not unique to Quebec. MH reform in Belgium also required that networks implement several new services without providing detailed directives or outcomes to stakeholders responsible for implementation [10].

Efforts to implement shared-care in the Quebec MH Reform were hampered by the absence of GPs in most HSSC-MH adult primary care teams, as well as their general lack of interest in taking on clients with MHD. Moreover, while GPs in medical clinics ranked second in terms of frequency of interaction with HSSC-MH primary care teams, they were sixth in terms of satisfaction, which suggests something lacking in terms of continuity of care or quality of services. This could be explained by the fact that GPs are also solicited for health issues other than MHD. Also, GPs have little time to provide psychotherapy, as consultation time is severely limited in primary care [46, 47]. Moreover, while GPs did treat common MHD, most avoided taking on clients with severe MHD or co-occurring MHD-SUD, referring them automatically to MH specialized services [48–50]. This finding corresponds to previous studies where the involvement of GPs with severe MHD or more complex cases was reportedly problematic [46, 51]. Recent MH reforms in Australia [8], and Finland [16]), reveal the central role of GPs as well as psychiatrists who were closely involved in the operation of multidisciplinary MH teams.

The HSSC-MH primary care teams treated common MHD, but also served an important minority of clients with severe MHD, personality disorders, suicidal ideation, and co-occurring MHD-SUD as well as chronic physical illnesses. The literature suggests a number of benefits accruing from primary care services for these clients: primary care is less stigmatizing than specialized MH services [52], fosters better community reintegration for individuals with severe and chronic MHD [33], and facilitates the treatment of chronic physical disorders often associated with MHD or SUD [53]. One may argue, nonetheless, that follow-up for some clients with severe or complex MHD may be more adequately provided by assertive community treatment teams, as highly specialized ambulatory services, rather than by primary care or intensive case management teams [54]. The referral of severe and complex cases, or less stabilized clients, to primary care may have resulted from the lack of specific eligibility criteria governing client referrals to primary versus specialized care, and the need to establish clear boundaries between the two levels of service.

Concerning the second objective (quality of care), the Quebec MH Reform prioritized the implementation of respondent-psychiatrists in order to enhance the expertise of primary care providers. The results show that respondent-psychiatrists collaborated more with HSSC-MH primary care teams than with GPs, however, and considered their impact higher on the quality of care provided by the HSSC teams. One likely explanation for this result was that respondent-psychiatrists had more previous contact with clinicians on HSSC-MH primary care teams, many of whom had been transferred from MH specialized services, than they had with GPs working in medical clinics. By contrast, according a recent Quebec study [49], 50 % of GPs had no previous contact with either psychiatrists or other MH professionals. Collaborative care is easier to implement when the providers involved have pre-existing relationships [47]. It should also be noted that respondent-psychiatrists were implemented late in the MH reform, suggesting the need for more experience with this strategy by both parties. Moreover, respondent-psychiatrists devoted very little time to GPs in terms of either telephone consultations or face-to-face discussions, which may explain their difficulties in building effective relationships with GPs. The implementation of successful innovation take time, especially initiatives like shared-care that entail a major shift in the MH system and in GP practices [55].

The Quebec MH Reform, unlike certain others [10], did not provide sufficient guidance on clinical processes, or on the use of clinical approaches or clinical/evaluation tools, which suggests why certain processes were underutilized. By contrast, MH reforms in countries like England [7], or Australia [8] provided clear guidelines around the implementation of clinical evaluation tools, best practices, and processes favoring the improvement of MH services, as well as performance assessment frameworks and specific indicators for MH system evaluation [15]. Curiously, certain strategies associated with co-occurring MHD-SUD services were more adequately implemented in the networks studied than MH strategies. For instance, screening and assessment tools for SUD were used more often than similar tools for MHD. Primary care teams often used motivational interviewing, which should be particularly effective for co-occurring MHD-SUD [56]. This may reflect the training offered to HSSC primary care teams by the Quebec Ministry from 2007, as part of its SUD program [57], that was generated by the high prevalence of co-occurring disorders in MH caseloads. Research suggests that half of individuals with a lifetime SUD may also have at least one lifetime MHD [58].

Concerning the third objective (continuity of care), referral procedures were the most successful integration strategy implemented. This result stands to reason as referral procedures represent the main form of inter-organizational collaboration, demanding the least amount of mutual dependence and organizational involvement [59]. Unlike referral procedures, service agreements formalize collaboration between organizations, and define their respective roles and responsibilities. Most HSSCs succeeded in formalizing service agreements with SUD rehabilitation centers for evaluation and collaborative treatment of individuals with co-occurring disorders, and with crisis centers for evaluation and follow-up of individuals at elevated risk for suicide. This seems to account for the high level of satisfaction among HSSC-MH primary care teams in their interactions with these organizations. In terms of liaison officers, participants cited their interactions with addiction specialist nurses as most satisfactory. The added value of liaison officers was underscored by the implementation of liaison teams to reduce the overflow of individuals with SUDs in emergency rooms in the context of the ministerial reform of the SUD program that was occurring simultaneously [57]. Working in partnership with emergency rooms and hospital units, the liaison teams systematically tracked clients with SUD or co-occurring MH-SUD and directed them to appropriate services [60]. According to a recent study, clients with SUD identified in emergency rooms by liaison nurses were 30 times more likely than others to enter a therapeutic program [61].

Another key network integration strategy, shared training, was moderately implemented, suggesting that the MH Reform did not view staff training as a priority, unlike reforms elsewhere [15]. While the training offered by the MH National Center of Excellence was important for implementation of intensive case management, other training opportunities for GPs and clinicians from HSSC-MH primary care teams were lacking. Yet good training infrastructure is essential to successful implementation of health reforms; training enhances provider competence and the appropriateness, quality and effectiveness of interventions [35], while also promoting a common vision [62, 63]. Unfortunately, training is too often perceived as a luxury, and may fall victim to budget cuts early on [64].

Finally, it is interesting to note that resource scarcity may be either a barrier or facilitator to reform, and to network integration [34]. According to resource-dependency theory, limited resources require organizations to work together [65]. The literature confirms however, that the extensive availability of MH services allows networks to introduce a greater variety of implementation strategies, such as standardized clinical evaluation tools, service agreements, shared-training, and the introduction of respondent-psychiatrists and liaison officers, all of which enhance service integration [34]. Other barriers to MH primary care consolidation and network integration reported by participants coincided with barriers described in the implementation literature [11, 38, 40–42]. An accumulation of hindering factors may have resulted in service duplication or compromised service continuity. Among these, resistance to change on the part of psychiatrists and GPs, staff turnover and lack of leadership were the most important barriers encountered. Strong local leadership as well as sustained training would be required in order to reduce resistance [66]. Despite significant support from the MH National Center of Excellence there was insufficient momentum for real transformation of the Quebec MH system to occur.

### Limitations

This study has a number of limitations. First, the results may not be generalizable to other countries, as the findings reflect characteristics of the networks and reforms studied. By the same token, generalization of the results across Quebec should be made with caution, despite efforts to provide a representative selection of networks. Second, study participants may have over- or under-estimated the actual degree of reform implementation or integration in their networks. In order to neutralize participant bias, the results were compared and validated by members of the research team in collaboration with the research advisory committee using a mixed-method approach and data triangulation. Finally, very few diverging viewpoints were noted among participants, tending rather to be more complementary or convergent.

## Conclusion

This is the first study to evaluate implementation of the Quebec MH Reform 2005–2015, highlighting the key strategies that underpinned successful network integration and lessons learned for other international MH reforms. The results demonstrate that primary care consolidation was greatly enhanced by the creation of HSSC-MH primary care teams and the integration of respondent-psychiatrists, with particular benefit for services to clients with common MHD. However, primary care services were not fully consolidated, as envisaged by the MH Reform, mainly due to the lack of operational mechanisms and protocols delineating the roles of new services and structures. The lack of GP input and relatively little time devoted to them by respondent-psychiatrists also hindered the consolidation of MH primary care services. In addition, the MH Reform fell short in terms of strengthening the implementation of best practices, formalizing network strategies, and providing support, training and performance indicators. The main advances identified in clinical practice were increased use of screening and assessment tools, and motivational interviewing techniques for individuals with co-occurring MHD-SUD. Yet, as indicated earlier, these advances may represent the indirect outcome of another reform of the SUD program that occurred simultaneously with the MH Reform.

The study findings suggest six recommendations for more successful implementation of the MH Reform, with potential relevance for MH reforms elsewhere. First, MH reform needs to focus not only on service implementation, but also on the development of network integration strategies and the provision of best practice guidelines. Second, systematic training programs on the use of clinical evaluation tools and clinical approaches for MHD need to be developed at the provincial level. Third, performance indicators specifying the desired results, and a greater commitment to the implementation of evidence-best practices, should be established. Fourth, the consolidation of existing HSSC-MH primary care teams should be completed, as well as the implementation of shared-care initiatives (respondent-psychiatrists). Fifth, the integration of GPs and psychiatrists into all HSSC-MH primary care teams needs to be prioritized, along with improved strategies to interest GPs in MH. Sixth, the improvement of integrated networks and a better continuum of care for clients with MHD depend crucially on the implementation of more formalized integration strategies to insure a better continuum of care for clients with MHD.

## Additional files

Additional file 1: Summary of the structure/main sections of the interviews guides and questionnaires. (DOC 80 kb)
Additional file 2: Representative quotations. (DOC 40 kb)

## Abbreviations

ACT: Assertive community treatment
GH: General hospitals
GP: General practitioners
HSSC: Health and social services centers
MH: Mental health
MHD: Mental health disorders
PH: Psychiatric hospitals
SUD: Substance use disorders
