# Supplementary material for: Implementation of the Quebec mental health reform (2005–2015)
Source: BMC Health Serv Res. 2016 Oct 18;16:586. doi: 10.1186/s12913-016-1832-5 (PMC5069811; doi:10.1186/s12913-016-1832-5)
Supplement: Additional file 1: — Summary of the structure/main sections of the interviews guides and questionnaires. (DOC 80 kb) [file 12913_2016_1832_MOESM1_ESM.doc]

**Additional file 1: Interviews guides and questionnaires**

**Interview guides (sections/main topics)**

1. **Regional managers, primary care teams managers and senior hospital executives**
2. **Presentation:**
3. **Current role, and level of decision-making responsibility in your organization, and in the service network?**
4. **Client characteristics:**
5. **How would you describe the main needs and challenges of yours clients with the following types of conditions:**

- **Common mental disorders?**
- **Serious mental disorders?**
- **Co-occurring disorders?**

1. **Transfer of clients from specialized services to primary care:**

- **Please comment on how this process unfolded, and its effects on the organization of your services.**

1. **Implementation of the mental health reform:**
2. **What have been the impacts of deploying the mental health reform in your organization?**
3. **Does the change generated by the reform seem to have aimed at the best targets?**
4. **Mental health network integration**
5. **How would you consider that your network responds, or not, to the needs of the population?**
6. **What partnerships, formal and informal, exist at present within the network?**
7. **What are the mechanisms of governance operating within the network?**
8. **What is your overall evaluation of the strengths, or potential, of your network?**
9. **Facilitators and barriers to implementation and to network integration**
10. **Please describe the principal issues and factors that have emerged as facilitators or barriers to implementation of new structures in the context of the mental health reform.**
11. **What were the main challenges encountered in implementing the respondent-psychiatrist function and HSSC-MH primary care teams in your territory?**
12. **Recommendations for improving mental health services**
13. **What are your recommendations for improving the integration of your services in order to improve mental health services in your territory?**
14. **Is there anything you can add or is there any point for discussion that may have been omitted?**
15. **Respondent-psychiatrists**
16. **Presentation:**

**1. For how long have you been a respondent-psychiatrist?**

1. **Client characteristics:**
2. **Please describe for us the types of patients that you treat.**
3. **Among the patients served, are there priority patients, or those targeted in particular by respondent-psychiatrists? Which patients?**
4. **What is the importance of your contribution in terms of specific support for patients with comorbid mental health/substance use disorders?**
5. **What are the principal needs of patients seen in your services, and what are your main challenges in responding to them?**
6. **Organization and integration of adult mental health services**
7. **How do you perceive the roles, responsibilities, and functions of the respondent-psychiatrist, and how have these functions evolved since implementation of the reform?**
8. **In the context of your functions as respondent-psychiatrist, please describe for us, if necessary, the interactions between you and different teams or professionals, and please identify the strengths and limitations of these interactions.**
9. **Can you identify any interactions that are missing, where your services would be needed?**
10. **Overall, how would you qualify the level of diffusion of shared-care in your network, and could you describe the evolution of shared-care since the reform?**
11. **What are the impacts of your work on strengthening the competencies of general practitioners or other mental health professionals?**
12. **What is your assessment of your role as a respondent-psychiatrist? a) by other healthcare professionals?, b) by your fellow psychiatrists, as well as c) by your organization?**
13. **How could your functions be optimized in order to improve the response to the needs of patients with mental health problems in the network?**
14. **What is your assessment of the strengths or potential of your network?**
15. **What in your opinion are the elements that would improve overall performance in the organization of mental health services in your LSN?**
16. **Is there anything you can add or is there any point for discussion that may have been omitted?**
17. **General practitioners**
18. **Presentation:**
19. **Current role, with regard to mental health specifically.**
20. **Client characteristics:**
21. **How would you describe the main needs and challenges of yours clients with the following types of conditions:**

**• Common mental disorders?**

**• Serious mental disorder?**

**• Co-occurring disorders?**

1. **Please describe for us the typical types of patients for whom you provide mental health services since implementation of the reform.**
2. **Describe for us the typical types of cases for whom you would need support from the mental health network, and those for whom you don`t receive support.**
3. **Organization and integration of adult mental health services for the optimization of treatments offered to your patients:**
4. **Describe for us the “traditional support” that you were receiving for mental health services before implementation of the reform.**
5. **Describe for us the support services that you receive presently in the context of shared-care or other forms of collaboration.**
6. **What is your assessment of the support procedures aimed at improving treatment for adults with mental health problems?**
7. **What is your assessment of these support procedures in terms of improving your competencies or those of your colleagues?**
8. **What in your opinion are the elements that would permit an improvement in the overall performance of mental health service organizations in your local service network (LSR) in order to provide better healthcare for adults suffering from mental health problems?**

**D. Is there anything you can add or is there any point for discussion that may have been omitted?**

1. **Directors of Community organizations**

**A. Presentation**

**1. Current role, and the decision making capacity of your organization in the network.**

**B. Client characteristics:**

**1. How would you describe the main needs and challenges of yours clients with the following types of conditions?**

**• Common mental disorders?**

**• Serious mental disorder?**

***•* Co-occurring disorders?**

**2. Do you perceive any changes since implementation of the reform in terms of the client profile in your services or in terms of ridership and revenues?**

**C. Implementation of the mental health reform:**

**1. How did the mental health reform respond to the expectations of community organizations?**

**2. What have been the impacts of the reform process on the consolidation and coordination of community organizations?**

**3. Does the change generated by the reform seem to have aimed at the best targets?**

**D. Mental health network integration:**

**1. How would you consider that your network responds, or not, to the needs of the population?**

**2. What are the current partnership links between community organizations and other adult mental health resources in the network?**

**3. With which partners do you have formal ties (e.g. liaison mechanisms between organizations)?/ informal ties?**

**E. Facilitators and barriers to implementation and to network integration**

**1. Describe for us the main issues encountered, and the factors that emerged as facilitators or barriers in the implementation of new structures since the mental health reform?**

**2. What factors facilitate or impede relationships between partners?**

**F. Recommendations for improving mental health services**

**1. What are your recommendations for improving the integration of services?**

**What are your recommendations with a view toward improving the overall performance of your network?**

**G. Is there anything you can add or is there any point for discussion that may have been omitted?**

**Questionnaire (sections/mains topics)**

1. **Managers (Primary care)**
2. **Individual characteristics (age, gender, position, seniority, etc.)**
3. **Client characteristics**
4. **Age**
5. **Mother tongue**
6. **Income**
7. **Diagnosis**
8. **Other clinical variables (suicidal ideation, problems with justice system, high services users, etc.)**
9. **Utilization of services during the last 12 months (general practitioner, psychologists, emergency, etc.)**
10. **% clients referred to other services (specialized services, mental health community organizations, etc.)**
11. **Team profiles**
12. **Number of professionals in the team (psychiatrists, general practitioners, nurses, psychologists, social workers, psycho-educators, occupational therapists, substance use disorder specialists)**
13. **Clinical activities**
14. **% time allocated per week to evaluation, treatment or intervention**
15. **Case load per professional**
16. **Frequency of client follow-up**
17. **Duration of client follow-up**
18. **Frequency of clinical approaches used (stepped care, cognitive behavioral approach, motivational interviewing, strengths model, care pathways, recovery approach, self-management)**
19. **Frequency of clinical evaluation tools used (screening tools for mental health disorders, screening tools for substance use disorders; assessment tools for mental health disorders, assessment tools for substance use disorders; client satisfaction assessment tools, protocols and best practice guidelines)**
20. **Level of implementation re network integration strategies (liaison officers, shared training, shared staff, service agreements, network resource directories, organizational referral procedures, interorganizational referral procedures, shared clinical records, substance use disorder specialists, respondent-psychiatrists)**
21. **Frequency and satisfaction of interactions involving network teams or organizations**

- **with other HSSC teams (one-stop services, general services, intensive case management, other)**
- **with specialized services (respondent-psychiatrists, emergency rooms, day hospitals, hospital units, assertive community treatment, out-patient clinics, other)**
- **with other network organizations (general practitioners in medical clinics, pharmacists, crisis centers, day centers, non-mental health community organizations, substance use disorder rehabilitation centers, other)**

1. **Respondent-psychiatrists**
2. **Individual characteristics (age, gender, position, institutional affiliation, seniority, etc.)**
3. **Client characteristics**
4. **Age**
5. **Mother tongue**
6. **Income**
7. **Diagnosis**
8. **Other clinical variables (suicidal ideation, problems with justice system, high services users, etc.)**
9. **Utilization of services during the last 12 months (general practitioner, psychologists, emergency, etc.)**
10. **% clients referred to other services (specialized services, MH community organizations, etc.)**
11. **Respondent-psychiatrist activities, and time allocations**
12. **Case discussions: a) with general practitioners in medical clinics; b) with mental health primary care teams**
13. **Diagnostic evaluations: a) with general practitioners in medical clinics; b) with mental health primary care teams**
14. **Treatment recommendations: a) with general practitioners in medical clinics; b) with mental health primary care teams**
15. **Pharmacological recommendations: a) with general practitioners in medical clinics; b) with mental health primary care teams**
16. **Other clinical activities: a) with general practitioners in medical clinics; b) with mental health primary care teams**
17. **Telephone consultations (hours per month): a) with general practitioners in medical clinics; b) with mental health primary care teams**
18. **Face to face consultations (hours per month): a) with general practitioners in medical clinics; b) with mental health primary care teams**
19. **Coordination with mental health specialized services (hours per month)**
20. **Impacts on mental health services:**
21. **On general practitioners in medical clinics: a) improvement in capacity to make mental health diagnoses; b) improvement in quality of patient case management; c) Improvement in the numbers of patients followed; d) improvement in the ability to orient patients toward appropriate services; e) improvement in cooperation with mental health primary care teams**
22. **On Mental health primary care teams: a) improvement in ability to evaluate patients; b) improvement in the quality of patient follow-up; c) improvement in the numbers of patients followed; d) improvement in the ability to orient patients toward appropriate services; e) improvement in cooperation with specialized mental health services;**
23. **Assessment of collaboration: a) with general practitioners in medical clinics; b) with mental health primary care teams**
