# Supplementary material for: Implementation of the Quebec mental health reform (2005–2015)
Source: BMC Health Serv Res. 2016 Oct 18;16:586. doi: 10.1186/s12913-016-1832-5 (PMC5069811; doi:10.1186/s12913-016-1832-5)
Supplement: Additional file 2: — Representative quotations. (DOC 40 kb) [file 12913_2016_1832_MOESM2_ESM.doc]

**Additional file 2: Representative quotations**

| **Factors that hinder or facilitate primary care consolidation**  *Protocol on how to operationalize measures of the MH Reform*: “The implementation of the one-stop services was done without any guidelines at the beginning of the Mental Health (MH) plan. (22- manager, Health and social service center (HSSC))”  "It would be nice if on the provincial level they would have a reference protocol for the one-stop services so that it is the same everywhere and more simple. (49- manager, HSSC)”  *Protocols that define team role and clientele*: "A protocol was elaborated in order to give structure to the MH primary care team and to define the clientele. They even met with other programs in the HSSC to better define the client profile that should belong to the MH primary care team. (70- manager, HSSC)”  *Lack of funding:* “Only half of the required staff for the mental health teams has been obtained, we have 23 full time staff while we would need 46…this is due to a lack of funds. (36- manager, HSSC)”  *Staff turnover:* “There is a lot of staff turnover for various reasons: retirement, a desire to try other things, sick leaves, and maternity leaves among younger staff. Replacements may lack knowledge, experience, and training. (29- manager, HSSC)”  *Experienced staff*: “ We have a very strong and solid team, our team is very motivated and clinicians have developed their own specializations over the years (69- manager, HSSC)”  *Resistance to change:* “Even when information about the one-stop services is provided to general practitioners, it is a message that is difficult for them to understand; there needs to be a lot of repetition in order to get them to adhere to the new organization of services. (74- manager, Regional agency)”  *Reluctance of general practitioners to use one-stop services:* Certain general practitioners still want to have direct access to psychiatric services… (39- manager, Regional agency)”  *Non-implication of general practitioners in HSSC-MH primary care teams: “*Recruitment of general practitioners is difficult for the mental health team because mental health is often a practice that is difficult for them (73- director, General hospital)”  *Lack of interest from general practitioners in mental health:* “There is only a minority of family doctors who have an interest and will agree to take on mental health patients. (34- psychiatrist)”  *Complex mental health disorders cases: “*Comorbidity with mental health and substance use disorders is becoming a constant, and is also seen in increasingly younger clients. (26- general practitioner)”  *Confusion between the respective roles of HSSC and MH community organizations concerning intensive case management:* “There is confusion with regard to the role of clinicians in the hospital and those in community organizations regarding who does intensive case management…. (73- director, General hospital)”  *Assessment tools:* “The HSSC-MH primary care team has been trained on the screening tool for substance use disorders. (31- manager, HSSC)”  *Lack of training:* “Staff training is a challenge particularly with so much turnover and younger staff. Human and financial resources are lacking for training (replacing absent staff on the job. It is sometimes impossible to release providers for a crucial 4-day basic training which is necessary in order to meet the quality standards for clinical practice…. (33- manager, HSSC)”  *Support/Coaching from the Mental Health National Centre of Excellence:* *"*Coaching was done by the MH National Center of Excellence which helped clinicians to understand the philosophy of the intervention, their role and the clientele that they needed to reach. (76- psychiatrist)”  **Factors that hinder or facilitate interaction between primary care and specialized services**  *Reluctance of general practitioners to consult respondent-psychiatrists: “*There still remains a negative perception from general practitioners about the usefulness and relevance of respondent psychiatrists. (41- general practitioner)”  “General practitioners are not compensated for meetings with psychiatrists whereas psychiatrists are well compensated. (40- manager, HSSC)”  *Reluctance of certain psychiatrists to assume the function of respondent:* “Psychiatrists who take on the function of respondent-psychiatrist are afraid that their wait lists will increase because the goal in the family medicine groups is to help general practitioners. (76- psychiatrist)”  *Liaison officers: “*We have put in place a mental health liaison nurse in the emergency department here. After triage, there will be another triage by this nurse to see if we can avoid a consultation in psychiatry… We also have a specialized liaison nurse from the addiction rehabilitation center at the emergency room … (27- manager, General hospital)”  *Service agreements:* “We have more and more of them. We have some with community organizations, we have some with the HSSC, we have some with the psychosis program, we have all kinds of service agreements. (02- director, Psychiatric hospital)”  *Shared-training:* “There is an “à la carte” training; the people who offer the training come from our team but it can be given to respondent-psychiatrists, to general practitioners, or offered to anyone in the network….. This fosters collaboration in the network… (12-manager, HSSC)”  *Referral mechanisms:* “There is the form for accessing the one-stop services… at the beginning the form had a lot of questions, it was never-ending. Now, we have a questionnaire that is short and sweet. (10- nurse, Family medicine group – medical clinic)”  *Frequency of interactions:* “There is a monthly training at the family medicine group and bi-monthly at the one-stop services which is greatly appreciated. It has a good effect on collaboration. (12- manager, HSSC)”  *Satisfaction with interactions:* “The respondent-psychiatrist gave a “plus” to primary care… Primary care services don’t feel abandoned. They feel supported and therefore able to take on more clients. Also, contacts and communication with specialized services are easier. (68- manager, Regional agency)”  *Clarity in the respective roles of primary care and specialized services*: “There is confusion with regard to the role of hospital staff and community organizations about who is offering intensive case management. (73- director, General hospital)”  *Lack of communication between organizations: “*There are communication problems therefore creating poor service continuity in the network…The hospital doesn’t know what services are offered by the HSSC; neither do they know the service trajectory… (28- manager, Regional agency)”  *Hospital-centrism*: “Because we have a strong hospital-centrism in our region, the patient is not at the center of practice. (38- general practitioner, medical clinic)”  *Lack of funding*: "Resource scarcity actually obliges the partners to communicate with each other, to collaborate and to better define their respective responsibilities. (18- manager, Psychiatric hospital)”  *Patient-centered philosophy based on client needs:* “With the MH Reform out, we decided with the community organizations that they were going to stop doing case management, so they could focus on community support. There was a collective will to do things right and the rationale behind it was the client’s needs. (31- manager, HSSC)”  *Knowledge of other organizations and acknowledgment of their expertise: “*Psychiatrists and general practitioners now know about the HSSC services and they use them; they have become better acquainted with the people at the HSSC and have more trust. (56- manager, HSSC)”  *Proximity of organizations:* "One of the advantages in our network is that the entire service continuum (first line and specialized services) is in the same building, therefore close by each other; we are a semi-urban network where people know each other more and it is easier to create bonds. (68- manager, Regional agency)”  *Organizational apprehensions about developing service agreements with public institutions*: “They say all the time that they do not have money, but they are coming to us to sign service agreements. That's my fear: are we going to be the servants in the network; because they have to make cuts and then they're going to underfund what they will no longer be able to do? (37- manager, Community mental health organization)”  *Leadership:* "The role of the HSSC in the local coordination of services is more and more important. We have one employee who is responsible for the committees that were set up to promote collaboration in the network; with the creation of this committee there was a will to collaborate. (36- manager, HSSC)”  HSSC: Health and social service center  MH: Mental health |
| --- |
